# Assessment of the relationship between stress and performance in candidates for the Brazilian Bar Association examination

**DOI:** 10.1590/2237-6089-2019-0002

**Published:** 2020-12-04

**Authors:** Fernando E. M. José, Claudia W. Golbert, Laureane H. A. José, Emmanuelle T. Dallegrave, Christian H. Kristensen

**Affiliations:** 1 Pontifícia Universidade Católica do Rio Grande do Sul Porto AlegreRS Brazil Pontifícia Universidade Católica do Rio Grande do Sul (PUCRS), Porto Alegre, RS, Brazil.; 2 Universidade Federal do Rio Grande do Sul Porto AlegreRS Brazil Universidade Federal do Rio Grande do Sul (UFRGS), Porto Alegre, RS, Brazil.; 3 Centro de Estudos da Família e do Indivíduo Porto AlegreRS Brazil Centro de Estudos da Família e do Indivíduo (CEFI), Porto Alegre, RS, Brazil.; 4 Centro Universitário Metodista Porto AlegreRS Brasil Centro Universitário Metodista (IPA), Porto Alegre, RS, Brasil.

**Keywords:** Stress, evaluation, performance, Brazilian Bar Association examination

## Abstract

**Objective:**

This study investigated stress and performance levels in candidates for the Brazilian Bar Association examination (Exame da Ordem dos Advogados do Brasil) in Rio Grande do Sul, Southern Brazil.

**Methods:**

The following instruments were used: A sociodemographic data sheet, Lipp's Stress Symptom Inventory for Adults, the Ways of Coping Scale, the Adult Self-Report Scale, and the Self-Report Questionnaire. The final sample comprised 117 candidates, aged from 18 to 59 years (mean = 29.7, standard deviation = 7.8), 76 women (65%) and 41 men (35%).

**Results:**

In the first phase of the examination, 67 candidates were approved (57.3%), but there was no significant difference in terms of stress: stress symptoms were present in 76.1% of the successful candidates and 62% of the unsuccessful candidates; (χ^2^ (1) = 2.09; p = 0.148). In terms of stress phases, 70.6% of the successful candidates (n = 36) were in the resistance phase and 78.4% of these had psychological symptoms. The mean age of successful candidates (28.2 years) was lower than that of unsuccessful candidates (31.7 years); (t (115) = −2.48; p = 0.015). Attention deficit/hyperactivity disorder symptoms were detected in 18 successful candidates (26.9%) and 6 unsuccessful candidates (12.2%); (χ^2^ (1) = 2.85; p = 0.091).

**Conclusions:**

Candidates who were successful in the first phase of the Brazilian Bar Association examination tended to be younger and scored higher for attention deficit/hyperactivity disorder symptoms, but their stress levels did not differ from those of unsuccessful candidates.

## Introduction

People are increasingly exposed to stressful situations due to the continuous transformations of society. Increased stimuli, such as professional and personal demands, make interpersonal relationships more and more intense, causing conflict, tensions and stress.^1^ Stress and its influence on relationships have been investigated since Hans Selye's^2^ pioneering research on general adaptation syndrome, in which external demands lead to a stress response that is linked, in some cases, to physiological or psychological changes in the body that can negatively affect homeostasis.^3^

Since Seyle, further research has explored stress and its variables, adding new aspects such as coping response to explanatory models. From a theoretical perspective, stress and coping have been more valued in health psychology due to the interaction between the organism and the external environment.^4^^,^^5^ In the interactive stress model, coping refers to cognitive and behavioral efforts to manage internal or external demands or demands that are perceived to exceed the individual's personal resources.^6^

According to World Health Organization data, 90% of the world's population is affected by stress, indicating an urgent need to better understand this phenomenon, which has taken on the proportions of a global epidemic.^7^

However, it is also known that appropriate levels of stress increase efficiency and improve performance.^8^ Thus, stress perceived as necessary and beneficial can have positive effects on performance. However, when these levels are exceeded, an individual's psychological energy is exhausted, exceeding the ability to adapt, thus impairing performance, which can result in the person feeling useless and devalued, without purpose or attainable goals.^9^^–^^11^ Specifically, the degree to which individuals experience stress in a performance scenario depends on their cognitive assessment of the situation and its demands, as well as their belief that they have the necessary resources to deal with it.^12^^,^^13^

The increasing number of unsuccessful candidates for the Brazilian Bar Association examination has raised questions about the reasons for these high failure rates. There is an increasing demand for preparatory courses for the examination, which is curious, since it indicates that candidates feel it necessary to study subjects that they have already been taught at university. Thus, in an effort to better understand the high failure rates, we investigated the relationship between stress and performance in candidates taking the Brazilian Bar Association examination.

Based on the assumptions outlined above, we hypothesized that a high level of stress, i.e. when an individual's adaptive capacity is overwhelmed, would be linked to poor performance and failure. To test this, we studied the stress level of candidates taking the Brazilian Bar Association examination and the ways they cope with it prior to the examination. This examination was instituted by Provision n° 81/96 of the Federal Council of the Brazilian Bar Association (Conselho Federal da Ordem dos Advogados do Brasil),^14^ stipulating that people with an undergraduate law degree must pass the examination to practice law. Specifically, we sought to assess the relationship between stress and first phase results and investigate candidates’ coping strategies.

## Methods

### Overview

The present study was of an applied nature, taking a quantitative approach to achieve descriptive objectives using a cross-sectional design.

### Participants

The convenience sample was non-probabilistic, comprising 117 candidates taking the Brazilian Bar Association examination in Rio Grande do Sul, Southern Brazil. The sample was recruited from two preparatory courses in the city of Porto Alegre. The sample included candidates of both sexes, aged from 18 to 59 years old, who had undergraduate degrees in law and social sciences from any university, regardless of marital status, preparatory course schedule, or number of previous bar examination attempts, although some of these data were later controlled for, as shown in Table 1. Candidates who refused to participate in the study were not assessed.

**Table 1:** Sample characteristics

Characteristics		n = 117
Age (years), mean ± SD		29.7 ± 7.8
Sex		
	Male	41 (35.0)
	Female	76 (65.0)
Psychiatric medication		
	Yes	22 (18.8)
	No	95 (81.2)
Time of day of preparatory course classes*		
	Morning	46 (41.8)
	Afternoon	1 (0.9)
	Night	54 (49.1)
	Morning and afternoon	5 (4.5)
	Morning and night	3 (2.7)
	Afternoon and night	1 (0.9)
Number of previous examination attempts,† median (P_25_-P_75_)		2.5 (1-4)
	0	13 (11.4)
	1-2	44 (38.6)
	3-5	41 (35.9)
	> 5	16 (14.0)

Data presented as n (%), unless otherwise specified.

P_25_-P_75_ = interquartile range; SD = standard deviation.

* Seven participants (6.0%) did not answer this question.

† Three participants (2.6%) did not answer this question.

### Instruments

#### Sociodemographic data sheet

The sociodemographic data sheet covered the following variables: sex, age, marital status, number of previous examination attempts, college of origin, the preparatory course attended and the time of day classes were held, and use of psychiatric medications (psychotropic drugs).

#### Lipp's Stress Symptom Inventory for Adults (LSSI)^15^

This instrument objectively identifies whether the patient has stress symptoms and their type (somatic or psychological), as well as the phase of stress.

The LSSI consists of three tables: the first table covers symptoms from the last 24 hours, the second table covers symptoms from the last week, and the third table covers symptoms from the last month.

#### Adult Self-Report Scale (ASRS)^16^

This instrument consists of 18 items assessing attention deficit/hyperactivity disorder (ADHD), focusing on Diagnostic and Statistical Manual of Mental Disorders, 4th edition (DSM-IV), criterion A symptoms modified for adults, since several of the original items concern behaviors typical of childhood or adolescence (for example, “running and climbing”). The ASRS offers five response options indicating the frequency of symptoms: never, rarely, sometimes, often, and very often. Cutoff points have not been defined for Brazil and the scale only assesses ADHD criterion A; it does not determine whether the symptoms began before the age of 7 or whether they have affected at least two areas.

#### Ways of Coping Scale (WCS)^17^

This instrument contains 45 items divided into four factors: problem-focused coping (18 items), emotion-focused coping (15 items), search for meaning in religion (7 items), and search for social support (5 items). Answers are given on a five-point Likert scale (from 1 = I never do this; to 5 = I always do this). Scores range from 1 to 5; the higher the score, the greater the use of a given coping strategy.

#### Self-Report Questionnaire (SRQ)

This instrument, developed by Harding et al.,^18^ and validated in Brazil by Mari and Williams,^19^ identifies psychiatric disorders at the primary care level. It consists of 20 questions designed to detect “neurotic” disorders, currently called common mental disorders. To be considered as a possible case, the respondent must score seven or more affirmative responses (yes), which are worth one point each. This cut-off score was obtained by determining the questionnaire's sensitivity, specificity, and positive and negative predictive values in other samples and enables us to separate subjects into two groups: those likely to have a common mental disorder and those likely not to have one.

### Data collection procedures

This study was approved by the Institutional Review Board (Comitê de Ética em Pesquisa) at the Pontifícia Universidade Católica do Rio Grande do Sul (CEP 11/05349: annex A). The sample was recruited from two bar examination preparatory courses in the city of Porto Alegre. The directors of the courses were contacted in advance to explain the purpose of the study and propose a joint research project. After the directors accepted, classes of bar examination students were selected. At the classes selected, the researcher and a course staff member presented a general explanation of the study's research objectives and invited students to participate shortly thereafter.

Volunteers filled out an informed consent form, which detailed the study's objectives and procedures, guaranteeing their confidentiality and emphasizing the voluntary nature of their participation.

The instruments described above were administered in the preparatory course classrooms, which took approximately 45 minutes. Each candidate was provided with an envelope containing a Sociodemographic Data Sheet and the LSSI, WCS, ASRS, and SRQ, which were administered in that order according to their standard administration times. This occurred fifteen days prior to the bar examination. Data on the examination results were provided by the course directors, and the names of participants who passed were double-checked on the website of the Brazilian Bar Association of Rio Grande do Sul (Ordem dos Advogados do Brasil – Rio Grande do Sul).

After conclusion of the assessments, participants were invited to an interview to discuss the results.

### Data analysis procedures

Participants’ responses were collected and the results were input into a database. Data analysis was conducted using the Statistical Package for the Social Sciences (SPSS) 17.0. Quantitative variables were expressed as mean and standard deviation or median and interquartile range. Categorical variables were expressed as absolute and relative frequencies. Student's *t*-test was used to compare means between independent samples. The Mann-Whitney test was employed for variables with asymmetrical distribution. Pearson's chi-square test was used to assess associations between categorical variables. Multivariate Poisson regression was used to control for confounding factors and determine which factors were independently associated with passing the first phase of the bar examination. Prevalence ratios (PR) with 95% confidence intervals (95%CI) were calculated to assess the effect of each factor. Statistical significance was set at 5% (p ≤ 0.05).

## Results

Of the total sample (n = 117), 57.3% (n = 67) passed the first phase examination, while the remaining 42.7% (n = 50) failed. Table 2 shows that the only significant association between sample characteristics and success was with age, i.e., younger participants were more likely to pass the first phase.

**Table 2:** Associations between demographic variables and success in the first phase of the Brazilian Bar Association examination

Characteristics		Passed (n = 67)	Failed (n = 50)	p
Age (years), mean ± SD		28.2 ± 7.5	31.7 ± 7.8	0.015
Sex				0.243
	Male	20 (29.9)	21 (42.0)	
	Female	47 (70.1)	29 (58.0)	
Psychiatric medication				0.666
	Yes	14 (20.9)	8 (16.0)	
	No	53 (79.1)	42 (84.0)	
Time of day of preparatory course classes				0.488
	Morning	29 (44.6)	17 (37.8)	
	Afternoon	0 (0.0)	1 (2.2)	
	Night	30 (46.2)	24 (53.3)	
	Morning and afternoon	4 (6.2)	1 (2.2)	
	Morning and night	1 (1.5)	2 (4.4)	
	Afternoon and night	1 (1.5)	0 (0.0)	
Number of previous examination attempts – median (P_25_-P_75_)		2 (1-4)	3 (1.5-5)	0.177

Data presented as n (%), unless otherwise specified.

P_25_-P_75_ = interquartile range; SD = standard deviation.

The variables retained in the Poisson regression model constructed to assess which factors were independently associated with passing the first phase of the examination were age (p = 0.013) and ASRS score (p = 0.021). Candidates under the age of 30 had a 70% higher success rate than those over 30 (PR = 1.70; 95%CI: 1.12-2.60), while those who scored one point higher in total ASRS score (which could indicate ADHD symptoms) had a 5% higher success rate (PR = 1.05; 95%CI: 1.01-1.09).

There were no significant differences between candidates who passed or failed the examination in terms of presence of stress, mean total LSSI score, or stress phase. However, there was a significant difference in symptom types: psychological symptoms predominated among successful candidates (Table 3). Similarly, no differences in coping strategies were observed between successful and unsuccessful candidates.

**Table 3:** Association between LSSI and Ways of Coping Scale scores and success in the first phase of the Brazilian Bar Association examination

			Passed (n = 67)	Failed (n = 50)	p
LSSI					
	Stress				
		Yes	51 (76.1)	31 (62.0)	0.148
		No	16 (23.9)	19 (38.0)	
	Total score, mean ± SD		15.9 ± 9.0	13.6 ± 9.6	0.190
	Phase*				0.467
		Alertness	4 (7.8)	3 (9.7)	
		Resistance	36 (70.6)	23 (74.2)	
		Near exhaustion	11 (21.6)	4 (12.9)	
		Exhaustion	0 (0.0)	1 (3.2)	
	Symptoms*				0.009
		Physical	11 (21.6)	8 (25.8)	
		Psychological	40 (78.4)	18 (58.1)	
		Physical + Psychological	0 (0.0)	5 (16.1)	
Ways of Coping Scale – standardized score (0-100), mean ± SD					
		Factor 1: Problem-focused coping strategies	65.3 ± 14.2	61.8 ± 17.6	0.227
		Factor 2: Emotion-focused coping strategies	41.4 ± 14.2	38.1 ± 13.4	0.214
		Factor 3: Religiosity\fanciful thinking	50.8 ± 18.5	50.7 ± 18.9	0.978
		Factor 4: Search for social support	45.3 ± 16.6	42.9 ± 16.4	0.439
	Predominant coping strategy				0.789
		Problem-focused	59 (89.4)	43 (86.0)	
		Emotion-focused	7 (10.6)	7 (14.0)	

Data presented as n (%), unless otherwise specified.

LSSI = Lipp's Stress Symptom Inventory for Adults; SD = standard deviation.

* Only for those who scored positive for stress.

Based on the association between psychiatric disorder symptoms according to the SRQ and possible symptoms of ADHD according to the ASRS, successful candidates were more likely to have ADHD symptoms than unsuccessful candidates (Table 4).

**Table 4:** Associations between psychiatric disorder symptoms (Self-Report Questionnaire scores) and ADHD symptoms (Adult Self-Report Scale scores) and success in the first phase of the Brazilian Bar Association examination

Scales		Passed (n = 67)	Failed (n = 50)	p
Self-Report Questionnaire (psychiatric disorders), median (P_25_-P_75_)		7 (5-12)	5.5 (3-10)	0.100
	Present	37 (55.2)	24 (48.0)	0.557
	Absent	30 (44.8)	26 (52.0)	
Number of often or very often responses on the Adult Self-Report Scale, median (P_25_-P_75_)				
	Part A	2 (1-4)	1 (0-3)	0.062
	Part B	3 (1-5)	1 (0-4)	0.042
	Part A + Part B	5 (3-8)	3 (1-6.5)	0.028
ADHD				
	Present	18 (26.9)	6 (12.2)	0.091
	Absent	49 (73.1)	43 (87.8)	

Data presented as n (%), unless otherwise specified.

ADHD = attention deficit/hyperactivity disorder; P_25_-P_75_ = interquartile range.

## Discussion

A total of 57.3% of the participants in this study passed the first phase of the Brazilian Bar Association examination. The examination consists of two phases: the first tests objective knowledge and the second involves essay questions. No studies have specifically analyzed success in the first phase of the examination, since the data are released only after the final phase. According to data from the Rio Grande do Sul chapter of the Brazilian Bar Association, only 14.15% of all candidates passed the final test in 2009.^14^ At the time of writing, the participants in the present study still did not know whether they had passed the second phase of the examination. Thus, the data are discussed considering this limitation.

Regarding age, it was found that the successful candidates were significantly younger than the unsuccessful candidates. One possible explanation for this finding could be the differences in cognitive performance associated with age. Although most studies compare contrasting age groups (e.g. younger vs. older), it is known that there is a decline in cognitive function beginning with the second decade of life. People can preserve and mentally recall what has already been experienced, from concepts to facts to sensations. In daily life, adults, especially older adults, have greater difficulty with memorization since fact recovery is impaired. However, other factors could also explain this age difference, such as genetics, environment, personality, etc.^20^

This study did not find significant differences between successful and unsuccessful candidates in the first phase of the Brazilian Bar Association examination in relation to the presence of stress, mean total LSSI score, or stress phase. However, there was a significant difference in symptoms among candidates who scored positive for stress: psychological symptoms predominated among successful candidates, which could be associated with the positive effects of stress. Stress is understood as a rupture of an individual's homeostasis due to aversive stimuli, as well as an intense physical reaction to an event, whether negative or not.^21^^,^^22^ Although the term stress has negative connotations, up to a certain level, depending on a series of processes including perception, tolerance and beliefs, it can actually be beneficial. There are positive responses to stressful stimuli, such as intellectual and emotional growth and development, which Selye^2^ called “eustress.” The effort to adapt generates a sense of personal fulfillment, well-being and satisfaction, even if it involves unexpected effort^9^^,^^11^^,^^23^ in tense but healthy situations.

Of those who passed the examination, 26.9% were likely to have ADHD symptoms, which indicates a positive association between ADHD symptoms and passing the test. This, however, could be understood as the physiological response of a “fighting” organism rather than as a symptom of a psychopathological disorder. In a review of stress, Selye^24^ stated that the body always seeks to adapt to the stressful event and uses large amounts of adaptive energy in the process. In reacting to the stressor, the organism modifies itself physiologically to cope with an attack, temporarily changing to “fight or flight” mode, which allows the organism to direct all of its energy toward threat management.^25^^,^^26^ If the stressor is not eliminated, the organism shifts into a resistance phase in which it tries to adapt by expending a great deal of energy preparing to defend against a longer attack. In such cases, passive reaction predominates.^26^^–^^28^ Symptoms that can occur during this period include: arterial hypertension, social isolation, decreased productivity and creativity, tiredness, decreased libido, and attention and memory difficulties.^10^^,^^29^ In our study, 70.6% of the successful candidates were in the resistance phase, and it is possible that physiological and psychological changes associated with this phase could have led to higher ADHD symptom scores.

Interest in the relationship between stress and performance has a long history. In 1908, Yerkes & Dodson^30^ were the first to measure this relationship, resulting in the Yerkes-Dodson law, which states that the positive relationship between efficiency and performance does not extend indefinitely. In situations that demand constant adjustments, stress reaches a tolerance limit that contributes to decreased performance, efficiency, and even health. Thus, although effects psychologically perceived as necessary and beneficial can improve performance, when these levels are exceeded an individual's psychological energy is exhausted, exceeding the ability to adapt, thereby impairing performance, which can lead to feeling useless and devalued, with reduced capacity and unattainable goals.^9^^–^^11^ It can be inferred that this study's sample fits this relationship between stress and performance, since 76.1% of the successful candidates presented stress and psychological symptoms predominated in 78.4% of these cases.

Stressful events alone cannot determine an individual's stress level; each person faces them differently. An individual's stress level depends on subjective assessment and interpretation, which involve behavioral and cognitive responses.^31^^,^^32^ During the assessment process, people tend to interpret situations as more dangerous than they really are. In such cases, the stressor consists of thoughts, which, depending on the beliefs and norms acquired through life experiences, can interpret a new event as threatening.^33^^–^^35^

Specifically, the degree to which people experience performance-related stress depends on a decision, a cognitive assessment of the situation's demands and whether they believe they have the necessary resources to deal with it.^12^^,^^13^ Thus, many candidates for the bar examination may have negative beliefs and cognitive distortions about it, which are influenced by a number of factors. The considerable increase in demand for preparatory courses, many of which are offered by the universities themselves, makes us question the quality of education in Brazil, although we will not discuss this here, since it is beyond the scope of this study. However, these and other causes lead to anxiety about the examination, and candidates undergo an arduous journey until they feel truly “fit” to pass.

## Conclusions

This study evaluated the relationship between stress and performance in a sample of Brazilian Bar Association examination candidates attempting to begin their careers as lawyers. The findings of this study indicate that there was not a negative relationship between stress and performance. Of those who passed the first phase of the examination, 76.1% had high stress, the majority of whom (70.6%) were in the resistance phase. A great amount of energy is expended in this phase to deal with the stressor, which could be related to the candidate's success, as well as to the increase in ADHD symptoms, given that, despite obtaining satisfactory results, candidates may present symptoms related to the physiological and psychological changes involved in this process. In such cases, it would be advisable to evaluate candidates with positive ADHD results after the examination process is over to clarify this relationship.

Five important limitations were identified in this study, the first being that by the end of the study the candidates still did not know whether they had passed the second phase of the examination.

The second limitation was that all of the participants were taking a preparatory course for the examination, which precluded analysis of individuals who did not do so.

Third, the time elapsed since the participant's graduation from college was not considered, which could have been a significant factor in distinguishing candidates, since the ability to recall content could be more compromised after a greater period of time had elapsed.

Fourth, regarding psychiatric medication, no specifications were made regarding the types of drugs used, and ongoing ADHD treatment was not considered. It was thus not possible to quantify this variable as an element of interference in candidate performance. Likewise, we did not determine whether the participants had a prescription for such medications, which is important since psychoactive substances (mainly stimulants) are commonly used to improve cognition in healthy individuals.

Fifth, we did not consider whether the participants were also employed or the number of hours in their shifts, which could have influenced their availability to prepare for the examination and, thus, their stress level.

Further studies of the relationship between dysfunctional thoughts, personal beliefs, stress, and performance are needed to determine which factors are actually associated with stress, since this study found a relationship, but could not determine whether internal or external factors were chiefly responsible for the participants’ increased stress levels.
